# Biomechanical evaluation of prophylactic reinforcement of the linea alba with a novel method of positioning and fixation of polypropylene mesh, in an animal model

**DOI:** 10.1590/acb405325

**Published:** 2025-07-18

**Authors:** Thiago de Almeida Furtado, Álvaro Cota Carvalho, Marcelo Lopes Furtado, Luiz Ronaldo Alberti, Diego Paim de Carvalho Garcia

**Affiliations:** 1 Hospital Felício Rocho – Departamento de Ciências Médicas – Belo Horizonte (MG) – Brazil.; 2 Universidade Estadual Paulista – General Surgery Department – Faculdade de Botucatu (SP) – Brazil.; 3 Universidade Federal de Minas Gerais – Santa Casa BH – Belo Horizonte (MG) – Brazil.

**Keywords:** Hernia, Ventral, Models, Animal, Incisional Hernia

## Abstract

**Purpose:**

To evaluate the effectiveness of a new format of prophylactic mesh, with a new method of placement.

**Methods:**

Rabbits were randomly distributed into four groups: control rabbits without laparotomy group; sutured rabbits (SR); light-weight mesh rabbits (LWMR); and heavy-weight mesh rabbits (HWMR). The meshes were cut to a size of 10 cm in length by 1 cm in width. A longitudinal, median abdominal incision, 11 cm in length, was performed, followed by an incision over the linea alba, encompassing the entire thickness of the abdominal wall. On the 91st postoperative day, the animals were killed for linea alba scar evaluation.

**Results:**

There was a significant difference among the evaluated groups concerning elongation, maximum load, and elasticity modulus. HWMR and LWMR were statistically different from controls regarding elongation. As for the elasticity modulus, SR presented a higher mean when compared to the others. Microscopic visual analysis showed greater fibrotic scarring with a larger amount of implanted material in HWMR.

**Conclusion:**

The reinforcement of scars with polypropylene mesh does not increase tensile strength when compared to the use of sutures. Mesh implantation results in scar remodeling with greater fibrosis, leading to increased elongation. However, midline reinforcement using only sutures has greater elasticity than the groups reinforced with mesh.

## Introduction

The median laparotomy is the most performed surgical access to the abdominal cavity, as it allows rapid access without injury to muscles, nerves, and vascularization of the abdominal wall. This approach involves opening the linea alba, which represents the intersection of the aponeuroses that envelop the rectus abdominis muscles. However, it is also the incision most frequently associated with incisional hernias^1^,^2^.

The correct technique for closing the abdominal wall is the primary factor responsible for preventing incisional hernias^3^.

Bellón et al.^4^ published a relevant article suggesting the choice of rabbits for studies of abdominal wall healing. The results of traction tests (biomechanical) of mesh in animal models available in the literature suggested the possibility of increasing the maximum load resistance of scars with mesh, compared to suture models.

Therefore, the aim of this study was to biomechanically evaluate the effectiveness of a new format of prophylactic mesh, with a new method of placement, in an animal model.

## Methods

This work was carried out following the recommendations of the International Standards for Animal Protection and the Brazilian Code of Animal Experimentation and was approved under protocol 099/11 by the Ethics Committee for Animal Use of the Universidade Federal de Minas Gerais (UFMG). This study was conducted at UFMG, at the Central Vivarium’s surgery laboratory from the UFMG’s Department of Medicine.

The experimental model consisted of male New Zealand rabbits, 3 months old and weighing over 2 kg. Thirty animals from the Experimental Veterinary Farm were studied (there was no criteria for the number of recruited animals performed previous this study). All rabbits were identified and housed in the Central Vivarium of the Medical School at UFMG, one animal per cage, in a suitable location with ventilation and lighting, and were provided with daily rabbit feed and filtered water *ad libitum*. The animals were weighed using a common scale (Filizola, São Paulo, SP, Brazil), with capacity of 10 kg and divisions of 100 grams, and were examined daily for any evident ectoscopic affection.

**Figure 1:**
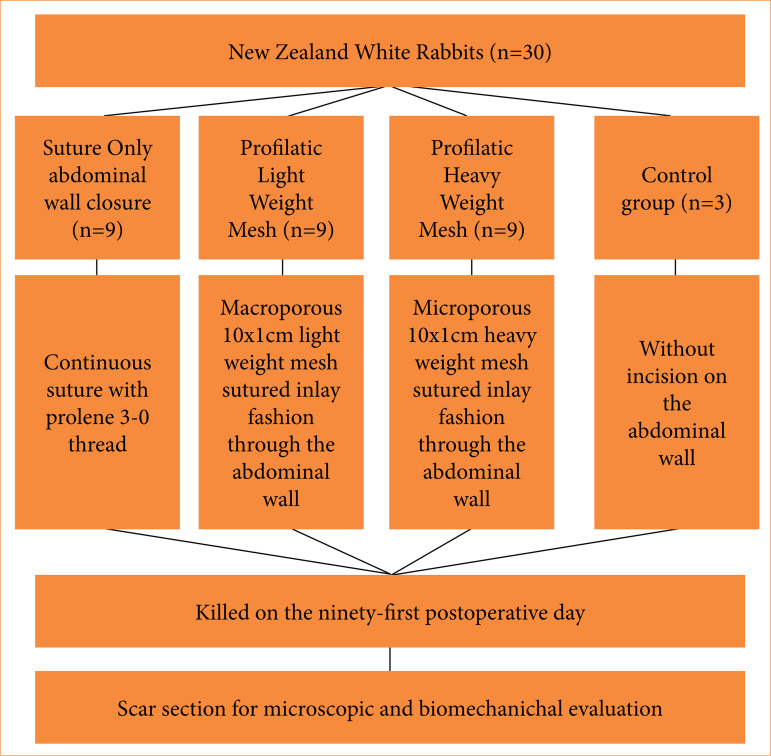
Flow diagram illustrating control and experimental groups allocated according to methods section.

Source: Elaborated by the authors.

The animals were then randomly distributed into four groups:

- Control rabbits without laparotomy (CRWL) (n = 3);
- Sutured rabbits (SR) (n = 9);
- Light weight mesh applied rabbits (LWMR) (n = 9);
- Heavy weight mesh applied rabbits (HWMR) (n = 9) (Fig. 1).

In this study, microporous polypropylene meshes of heavy weight (Bard Mesh, weight 105 g/m^2^, pore size 0.50 mm, Bard Inc., United States of America) were used in the HWMR. The other mesh used was a macroporous polypropylene light weight (Bard Soft Mesh, weight 44 g/m^2^, pore size 1.58 mm, Bard Inc., United States of America), utilized in the LWMR. The meshes were cut to a size of 10 cm in length by 1 cm in width.

The rabbits were anesthetized by intramuscular injection in the gluteal region with ketamine hydrochloride (Ketamin-S, Cristália, Itapira, SP, Brazil) at 5%, at the dose of 35 mg/kg (0.7 mL/kg), associated with xylazine hydrochloride (Rompun, Bayer, São Paulo, SP, Brazil) at 2%, at the dose of 6 mg/kg (0.3 mL/kg). When necessary, half of the initial dose of anesthetics was additionally administered to achieve adequate analgesia and sedation.

**Figure 2:**
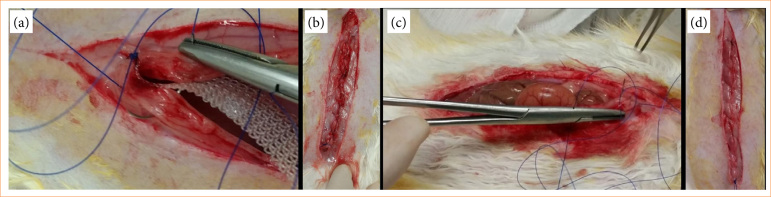
Aspects of experimental and control group of rabbits’ abdominal wall during and after reconstruction of the incised abdomen. **(a)** Mesh (10 × 1 cm) positioning throughout the incised abdominal wall for closure of rabbit’s abdomen. **(b)** Aspect of the closed abdominal wall with mesh positioned through the incision. **(c)** Sutured closure of the rabbits’ abdominal wall with 3-0 prolene thread. **(d)** Final aspect of rabbits’ closed abdominal wall with suture only.

Source: Elaborated by the authors.

The surgical procedure began with a longitudinal, median abdominal incision, 11 cm in length, encompassing the skin and subcutaneous tissue, followed by an incision over the linea alba, encompassing the entire thickness of the remaining abdominal wall, 10 cm in length. The closure of the surgical incision in the rabbit’s abdominal wall, according to each group, was performed with 3-0 polypropylene thread (Prolene, Ethicon, São Paulo, SP, Brazil) with primary approximation of the linea alba with or without prophylactic mesh implantation according to the group allocation, as depicted in Fig. 2.

**Figure 3:**
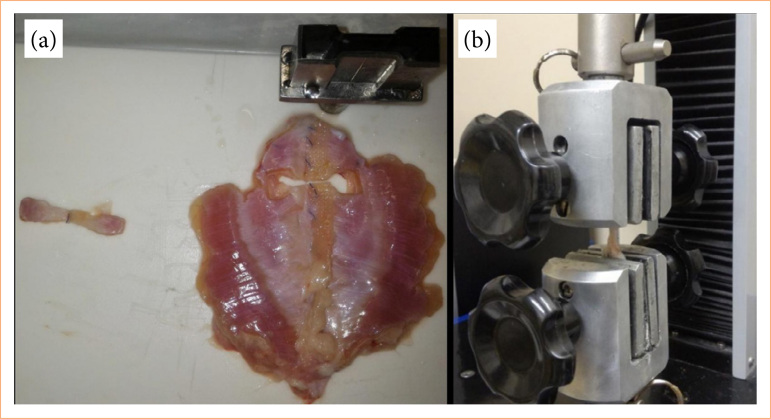
Source: Elaborated by the authors.

On the 91st postoperative day, the animals were killed for linea alba scar evaluation, through complete excision of the scar. Three or four identical test bodies were obtained from each abdominal wall specimen, depending on the excised wall’s size, using a specific knife. Tensile tests were conducted in 4 hours of animal death (Fig. 3).

Explanted abdominal wall of New Zealand rabbits and confectionery of test body being submitted to biomechanical test. (a) Test body explanted after excision of explanted abdominal wall of experimental group; (b) test body being submitted to tensile test of the scar.

Tensile tests were performed until rupture of each test body using a Shimadsu machine, model MWG, with capacity of 20 kN. Each end of the test body was grasped by a machine jaw, with the center of the body coinciding with the center of the distance between the jaws (Fig. 3).

One test body from each group was separated for histological analysis. After being embedded in paraffin according to routine methods, they were stained with Masson’s trichrome.

## Results

All variables showed normal distribution. Therefore, parametric tests were employed. Data homogeneity among groups was assessed using the Levene’s test. Maximum force and elasticity modulus exhibited different variances between groups. Hence, Dunnett T3 test was applied for mean comparisons. Analysis of variance (ANOVA) test was used for the comparison of variables such as elongation and maximum load. Multiple comparisons in ANOVA were conducted using Tukey’s test. The statistical analysis was single-blinded, as it was conducted by different researcher other than the surgeon that performed the procedures. In all tests, the significance level adopted was 5%. Therefore, comparisons with *p* ≤ 5% were considered significant (Fig. 1).

No animals died related to the surgical procedure or wound complications or incisional hernias during the three-month duration of the experiment. Maximum force did not differ between groups. There was a statistically significant difference among the evaluated groups concerning elongation, maximum load, and elasticity modulus (Table 1). HWMR and LWMR were statistically different from controls regarding elongation (*p* = 0.017 and *p* = 0.043, respectively). Both showed higher means than the control group (Table 1). Regarding elongation and maximum load, the HWMR exhibited a higher mean when compared to the others, but only the comparison with the control group was significant (*p* = 0.032).

Maximum force and elasticity modulus showed different variances between the groups. Therefore, Dunnett T3 test was applied for mean comparisons. For the comparison of variables elongation and maximum load, ANOVA test was used. Multiple comparisons in ANOVA were conducted using Tukey’s test.

**Figure 4:**
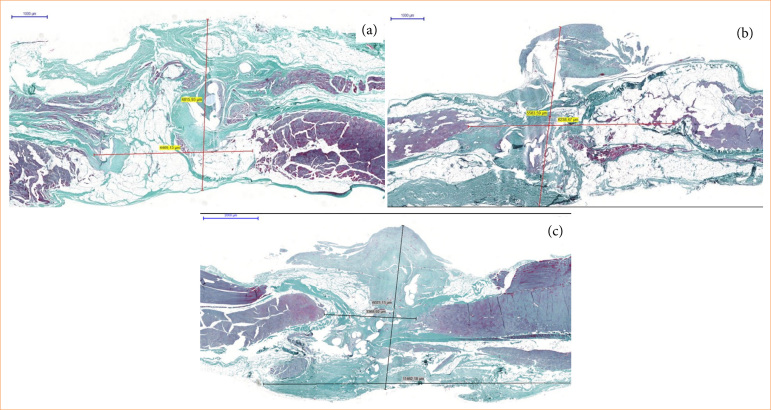
Histological analysis of test body’s scar and measurement of fibrosis through the midline, colored with Masson’s trichrome. **(a)** Suture only aspects of the scar with fewer collagen fibers. **(b)** Aspects of the scar in light-weight mesh, showing more collagen fibers formation the control group. **(c)** Heavy-weight mesh scar showing the most collagen deposition in the scar.

Source: Elaborated by the authors.

As for the elasticity modulus, SR presented a higher mean when compared to the others (heavy weight, *p* = 0.003l light weight, *p* = 0.003; and suture, *p* = 0.007). Microscopic visual analysis of slides in Masson’s trichrome showed greater fibrotic scarring with a larger amount of implanted material. In HWMR, the largest areas of fibrotic scarring were identified. LWMR presented less fibrosis, followed by SR, which showed the least fibrotic scars (Fig. 4).

**Table 1:** Results from biomechanical analysis of scar excised from New Zealand white rabbits showing variables of shear force (N), elongation (mm), maximum stretching force (mm.N), and elasticity modulus (N/mm).

Variables	Groups	Force (N)	Mean	Standard deviation	Confidence interval (95%)	*p* -value
Shear force (N)	Heavy weight	18	12.61	4.85	10.2–15.02	0.006
	Light weight	21	13.08	3.96	11.28–14.88	
	Suture	20	11.28	5.41	8.75–13.82	
	Control	13	16.85	8.96	11.44–22.26	
Elongation (mm)	Heavy weight	18	66.13	28.72	51.85–80.41	0.02
	Light weight	21	62.2	22.61	51.91–72.49	
	Suture	20	57.76	24.64	46.22–50.84	
	Control	13	39.05	19.51	27.26–50.84	
Maximum stretching force (mm.N)	Heavy weight	18	56.86	28.25	42.81–70.9	0.024
	Light weight	21	53.47	21.98	43.46–63.47	
	Suture	20	43.79	15.83	36.38–51.2	
	Control	13	34.67	18.16	23.7–45.64	
Elasticity modulus (N/mm)	Heavy weight	18	3.14	1.86	2.21–4.07	<0.001
	Light weight	21	3.07	1.8	2.25–3.89	
	Suture	20	4.12	2.65	2.87–5.36	
	Control	13	13.93	8.36	8.87–18.98	

Source: Elaborated by the authors.

## Discussion

There was no statistically significant difference in maximum force between groups. However, groups subjected to prophylactic implantation of low- or high-weight polypropylene mesh had greater elongation than control groups. In this context, the group subjected to implantation of prophylactic mesh with high-gram weight had a higher mean elongation and maximum load. Anyway, this data is only statistically significant when compared to the control group not subjected to laparotomy. Furthermore, the control group not subjected to laparotomy showed significantly higher mean elasticity compared to all other groups subjected to laparotomy, regardless the type of abdominal wall reconstruction (suture, light-weight mesh or heavy-weight mesh).

The reinforcement of the abdominal wall with polypropylene mesh is achieved by increasing tensile strength because of the fibrotic and scar tissue process. The biointegration of the mesh occurs through the infiltration of inflammatory cells and subsequently connective tissue. In our study, we observed that a higher density of polypropylene per material is associated with greater fibrotic scarring, especially compared to the absence of prosthesis adjacent to the scar. This result is consistent with other studies that evaluated the cellular response to polypropylene mesh^6^,^7^. Garcia et al.^8^,^9^, in different studies, assessed the inflammatory response to mesh presence, describing many giant cells consistent with a foreign body reaction in contrast to the predominant presence of polymorphonuclear cells in control groups or those repaired with sutures.

The use of prophylactic mesh is recommended in patients at high relative risk of developing incisional hernias, such as those undergoing conventional bariatric surgery or abdominal aortic aneurysm repair via laparotomy. Implantation in these cases is recommended in an onlay position over the scar, and in our study, we proposed the placement of the prosthesis adjacent to the incision (inlay). From our results, we can infer that, despite the scar being reinforced with a prosthesis, it does not achieve significantly higher values in terms of maximum tensile strength. This reinforces the findings of Hernani et al.^10^ in their study with mesh made in three-dimensional T shape, in which inlay mesh reinforcement, even with subsequent intraperitoneal reinforcement, does not lead to a significant increase in scar tensile strength in experimental models^5^,^10^.

Much debate exists regarding the benefit of using prostheses for the prevention of incisional hernias, mainly related to the absence of a standardized technique for the possible complications related to the prosthesis, such as surgical site occurrences and site infection. Our findings support the literature and lead us to believe that overlaying the mesh over the scar may be crucial for biomechanical reinforcement of the abdominal wall, as is done in hernia defect corrections; overlap can significantly increase scar tensile strength^11^–^13^. The lack of correlation between histology and biomechanical testing can be explained by the delay in the deposition of type-I collagen when there is a foreign body (mesh) present. The lower proportion of type-I collagen compared to type-III collagen leads to less stability of the scar, that is, a scar with a higher quantity of collagen fibers but less mechanical stability^7^.

Although our results are similar and supported by the literature, we believe that our sample may not be significant due to the small sample size in our experimental study. The use of rabbits is well-described and common in the literature, although large animals may provide a more similar analysis to human studies, and the most common models for studying diseases of the abdominal wall are performed with rats^14^–^16^.

## Conclusion

The reinforcement of scars with polypropylene mesh inlay does not lead to an increase in tensile strength when compared to the use of sutures for aponeurosis closure. Mesh implantation results in scar remodeling with greater fibrosis and collagen deposition, leading to increased elongation. However, the control group with midline reinforcement using only sutures had greater elasticity than the groups reinforced with mesh.

Investment in research that expands and consolidates knowledge about incisional hernia prophylaxis, especially regarding abdominal wall closure and prophylactic mesh use, and risk assessment, should be encouraged. Thus, it is necessary to establish the ideal characteristics of the mesh to be used for this purpose. This study contributes to this regard. Horizontal mesh overlap seems to be essential. Once these parameters are clearer with future studies, especially overlap size and mesh position, the development of a specific mesh for prophylaxis becomes possible, which can achieve the best clinical and biomechanical outcomes, specifically designed for incisional hernias prevention.

## Data Availability

Data sharing is not applicable.
